# Eye movements in response to different cognitive activities measured by eyetracking: a prospective study on some of the neurolinguistics programming theories

**DOI:** 10.16910/jemr.16.2.2

**Published:** 2023-05-16

**Authors:** Mathieu Marconi, Noelia Do Carmo Blanco, Christophe Zimmer, Alice Guyon

**Affiliations:** Université Côte d’Azur Neuromod- Mod4NeuCog,, France; Université Côte d’Azur Plateforme CoCoLab, France; Université Côte d’Azur- IPMC- CNRS UMR 7275, Valbonne, France

**Keywords:** Eye tracking, neuro-linguistic programming, rational thinking, gaze wandering, eye fixations, cognition

## Abstract

The eyes are in constant movement to optimize the interpretation of the visual scene by the brain. Eye movements are controlled by complex neural networks that interact with the rest of the brain. The direction of our eye movements could thus be influenced by our cognitive activity (imagination, internal dialogue, memory, etc.). A given cognitive activity could then cause the gaze to move in a specific direction (a brief movement that would be instinctive and unconscious).

Neuro Linguistic Programming (NLP), which was developed in the 1970s by Richard Bandler and John Grinder (psychologist and linguist respectively), issued a comprehensive theory associating gaze directions with specific mental tasks. According to this theory, depending on the visual path observed, one could go back to the participant's thoughts and cognitive processes. Although NLP is widely used in many disciplines (communication, psychology, psychotherapy, marketing, etc), to date, few scientific studies have examined the validity of this theory.

Using eye tracking, this study explores one of the hypotheses of this theory, which is one of the pillars of NLP on visual language. We created a protocol based on a series of questions of different types (supposed to engage different brain areas) and we recorded by eye tracking the gaze movements at the end of each question while the participants were thinking and elaborating on the answer. Our results show that 1) complex questions elicit significantly more eye movements than control questions that necessitate little reflection, 2) the movements are not random but are oriented in selected directions, according to the different question types, 3) the orientations observed are not those predicted by the NLP theory. This pilot experiment paves the way for further investigations to decipher the close links between eye movements and neural network activities in the brain.

## Introduction

Vision is a dominant sense in humans, with the greatest number of visual receptors (approximatively 100 million photoreceptors in the retina) as compared to other senses, and a great surface of the cortex treating visual information. Eye movements are under the control of complex neural networks interacting with the rest of the brain. Acting on eye movements such as in EMDR (eye movement desensitization and reprocessing) can affect brain activity for instance reducing posttraumatic stress disorders (17), although EMDR effects can also be obtained without the eye movements (7). The other way around, the direction of the movements of our eyes could thus be influenced by our cerebral activity (imagination, internal dialogue, memory...). Thus, a precise cerebral activity could induce the gaze to move in a precise direction (a brief movement, which would be instinctive and unconscious).

The neurolinguistic programming (NLP) approach to communication, personal development and psychotherapy, was developed in the 1970’s by Richard Bandler and John Grinder's. NLP claims that there is a connection between neurological processes (*neuro-*), language (*linguistic*) and acquired behavioral patterns (*programming*). One of the NLP theories stipulates that there is a link between the gaze orientation and the type of cognitive activity, depending on whether it depends more on a visual, auditory or tactile activity, and whether it involves memorization or imagination. NLP theory developed a complete map of gaze orientation in specific directions or zones in relation to mental activity (24) supposed to be valid not only for a single participant but also to some extent within the population (although it could vary, for instance, for right- and left-handed people).

This hypothesis was tested in the 1980’s when it was emitted (4, 22). However, the tools to study the gaze at that time were rudimentary (participants were asked questions such as: "Which is the brightest room in your home?" and one or more observers noted the directions in which the subjects' eyes were pointing). Many other studies were conducted on the subject of eyes in NLP, with conflicting results, but few used objective tools (5, 9, 10, 11, 19, 26, 27, 28). To circumvent these methodological flaws and test the NLP hypotheses on gaze orientation, there is a real need to develop new methodologies.

Using the tools of the eye-tracking, which allow an objective measurement of eye movements, we developed a protocol to test these theories. Eye-tracking is a method that consists in seeing in real time where the gaze is directed. Eye tracking devices were first developed at the very beginning of the 20^th^ century (18) and their applications are diverse. They have been used in research, particularly in behavioral studies, for many years. With the help of an eye-tracking system, we intend to test or refute the veracity of one of the pillars of the NLP on visual language. We created a protocol in which we asked the participants a series of questions that we designed in order to promote cognitive activities related either to visual, auditive or tactile modality, and to recruit either their memory or imagination and recorded their gaze in response to these questions by eye tracking.

## Methods

This study was performed in the CocoLab (MSHS Sud-Est - Université Côte d’Azur).

### Participants

The group of participants consisted of 31 people, 23 women and 8 men, aged from 19 to 60 with a median age of 42, right handed. Participants were volunteers.

All participants signed a consent of participation (Supplementary data 1), after being informed (Supplementary data 2) and filled a form specifying 1) their age and gender, 2) if they were wearing glasses or contact lenses (participants with glasses were asked to remove them during the test and we checked that wearing contact lenses didn’t affect the data acquisition), 3) if they were right-handed as asked, 4) if they used right-to-left writing and if so how often (the theory we use on the spatialization of numbers considers that since childhood we are used to the fact that the progression system goes from left to right (writing, timeline ...), but for people who write from right to left the progression system might have been reversed). 5) Finally, we asked them if they had any particularity (for instance hearing impairment, fatigue, stress, dysgraphia, dyslexia, dyscalculic disorder, native language other than French, or anything that could influence the comprehension of the test or the data).

### Design

We created a protocol in Tobii Studio, in which the participants had to listen to successive questions, fixate a cross on an empty black screen, think about the answer while leaving their gaze wandering after disappearance of the cross, and answer the question after a tone. We chose an empty black screen to avoid visual distraction and to collect only internally driven movements.

We explained to the participants the course of the experiment and gave them instructions without mentioning that we would record and focus on their gaze. We installed them comfortably on a chair, and used some removable walls available at the laboratory to surround the screen to avoid the gaze to go too far from it. The room was quiet. Participants were equipped with headphones that isolated them from outside noise, through which they could hear the questions without being distracted by unexpected noises in the room. They had to concentrate on the screen while Tobii Studio protocol was running and follow the instructions. We recorded the data on a PC computer and saved them on an external disk at the end of each experiment.

We used two setups in order to record eye tracking from two participants at a time. We disinfected the material between each participant. The whole session lasted less than 1 hour.

To test our protocol before going to the NLP hypothesis, we did a preliminary control experiment testing data already published in the literature. When the subject is doing spatial geometry tasks, and thus working mainly with the right hemisphere, the gaze is usually going to the opposite direction, i.e. to the left. On the contrary, when doing language tasks (such as searching for synonyms), thus working mainly with language areas located in the left hemisphere, the gaze usually goes to the right (8). Similarly, as we culturally have a spatialized vision of the numbers on a horizontal line, with small numbers on the left and big numbers on the right, imagining a big number is usually brings the gaze to the right, while imagining a small or negative number is supposed to bring the gaze to the left (8).

We asked a first series of 40 questions to verify that our protocol was reliable (questions are available in Supplementary Data 3).

Questions for the second part of the experiment:

We then tested in an independent protocol of 60 questions the hypothesis that different cognitive processes could orient the gaze in different directions. We created and recorded a series of questions (available in Supplementary data 3). As a control, we have used questions that require little thought, therefore supposed to induce little or no eye movement.

**Figure 1:**
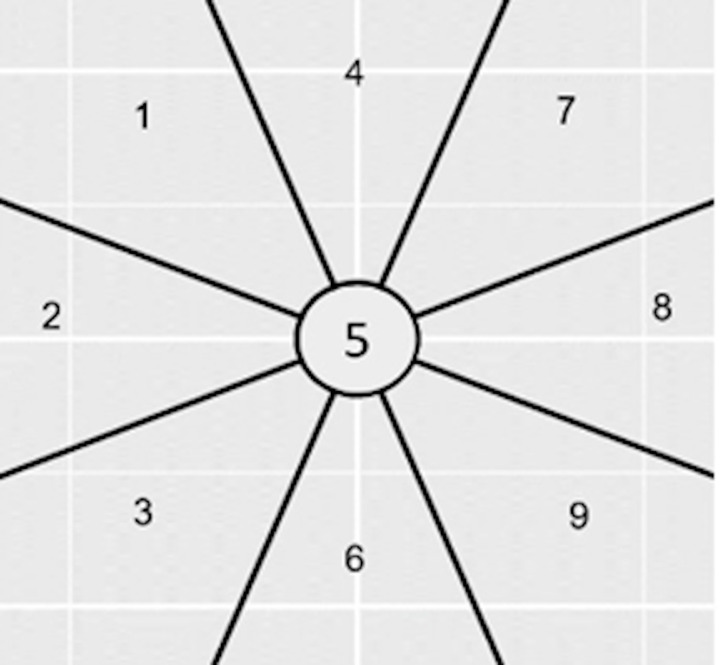
Visual representation of the NLP hypothesis to be tested. Eye positions that we used in the experiment, relative to the person.

Figure 1 illustrates the NLP hypotheses regarding gaze movements. Each number represents a gaze orientation supposed to be associated with a particular cognitive task in response to a certain type of questions.

The zones (relative to the person) are supposed to correspond to:

- 1 (Top left): Visual Remembrance

Example of question: What was the color of the shoes you were wearing yesterday?

- 2 (Left): Auditory remembrance

Example of question: What was your favorite song when you were a child?

- 3 (Bottom left): Internal dialogue

Example of question: Would you adopt an animal?

- 4 (Top): Internal visualization of a large picture object.

Example of question: How high are the posts in a rugby match?

- 5 (Center, no movement): Control question

Example of question: Do you have a driving license?

- 6 (Down): Internal visualization of a small but tangible picture object

Example of question: What is the size of a nail?

- 7 (Top right): Imagined visualization or projection into the future

Example of question: What would be your dream vehicle?

- 8 (Right): Sound construction

Example of question: Do you think you can create a melody?

- 9 (Bottom right): Kinesthetic feeling or emotionally charged experience

Example of question: What is the smell of your favorite perfume?

### Equipment

**Figure 2:**
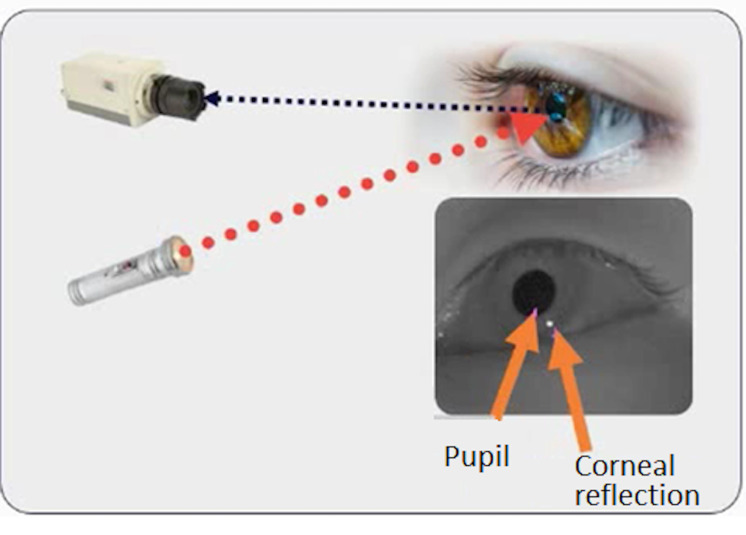
Principle of the eye tracker used in this study.

To record the gaze orientation, we used the system proposed by Tobii (Tobii Pro X3-120 eye-tracker). This eye tracker system is composed of an illuminator that emits infrared waves, a camera and a processor that analyses the information and converts the video into data (Figure 2).

The gaze precision of the eye tracker was 0.24° and the gaze accuracy was between 0.4° (in ideal conditions) and 0.7° (in the peripheral area of the screen). Data were post-processed with Pro-Lab.

### Material

Using Tobii Pro Studio software, we created two series of 40 and 60 questions in French in the form of two unique files. In order to avoid subjects' fatigue, we incorporated a break between the two protocols. Participants started by reading the instructions. Then, we calibrated the eye-tracking system. The eye tracking system was calibrated for each participant at the beginning of the sessions. Data were captured at a sampling rate of 120 Hz.

### Procedure

**Figure 3:**
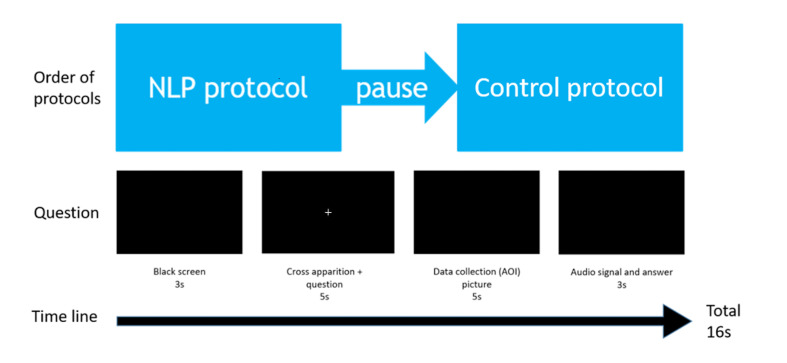
Scheme of the temporal succession of events occurring during the protocol

We created a set of 100 sequences of black screen with each question synchronized with the fixation cross, so that the starting point of the gaze would be the same for each question. For each sequence, the participant had to fixate a white cross (chosen to avoid having persistent images in the retina, background at 0%, cross pixels at 9% luminance relative to the white) while listening to the first question until the end. At the end of the question, the cross disappeared, allowing the gaze to move in any given direction (recorded by the software). Then an audio signal was given and the participant was asked to give orally the answer, which was recorded. A new question started, and so on until the end of the first series of questions (see Figure 3). 10 questions per condition were used for the control protocol and 6 questions per condition for the NLP protocol. Questions were presented in blocks of different question types in a random order in each block (Supplementary Data 3).

We took a short break (10 min) between the two series of questions.

Before starting the recordings, each participant was trained with a tutorial protocol to familiarize with the question format.

After creating the file to conduct the experiment, we defined the different areas of interest (AOI) for each video. Indeed, Tobii Pro Studio software can compute the data in order to give a series of parameters (such as the percentage of time spent in the AOI, the delay before the first entry in the AOI, the number of visits to the AOI, etc.) for each defined AOI that we are interested in.

We placed the AOI temporally just after the disappearance of the fixating cross, at the end of each question, for a total duration of 5 seconds. Tobii is constantly recording the fixations and the saccades. During the time of presence of the AOI, we selected the following parameters among the great number of parameters calculated by the Tobii software: Number of fixations of the AOI, Total fixation duration of the AOI, Time to first fixation of the AOI, Total duration of visits of the AOI, Number of visits of the AOI. We exported the data as an Excel format for analysis.

Data concerning the fixations preprocessed by Tobii (x and y coordinates of the fixations) were exported in R (version 4.0.3; 21) and we wrote a program aiming to determine the angle and the amplitude of each fixation vector relative to the center, which allowed us to refine the AOI.

Some of the participants had a lot of missing data for certain items (looked outside the screen). Data were removed from the analyses according to the following criteria: participants who had a rate of missing data greater than 10% in one of the two protocols (5 participants); participants who admitted having understood the nature of the test and having voluntarily fixated the screen randomly (1 participant); the fixations for which the reliability was very low according to Tobii (9.50% of the data “Validity_LR” column); and the items for which the last fixation occurring during the question was not in the control area (26.81% of the data, "is.lastfixasf.inside.control.area" variable). Indeed, one of the instructions we gave to the participants was to wait until the end of the question (i.e. wait until the cross disappears) before leaving the control area. We decided to exclude all the items for which the participant did not follow the instructions, as it was very important to analyze only the data that had the same starting point. In the end, we excluded 40.37% of the total of our data.

For the statistical analysis, we used a generalized linear mixed model (GLMM) to determine if the number of fixations in the central (control) area was more important in the control condition than in the other conditions. The mixed-model parameters were the following: 1 fixed-effect (i.e., condition), and 2 random intercepts (i.e., subjects and items).

To investigate if there was a relationship between the type of question asked and the zone in which the gaze is located, we used a chi² test. This allowed us to determine if the distribution of gaze fixations was purely random or if there were preferential directions. Our test consisted of establishing if the variables “Condition” (type of question asked) and “zone” (visited by the gaze) were dependent or not.

## Results

Results of the control experiment:

In this experiment, we used 10 questions per condition. We compared the data obtained with this protocol to the results of the control condition (collected during the NLP protocol).

**Figure 4:**
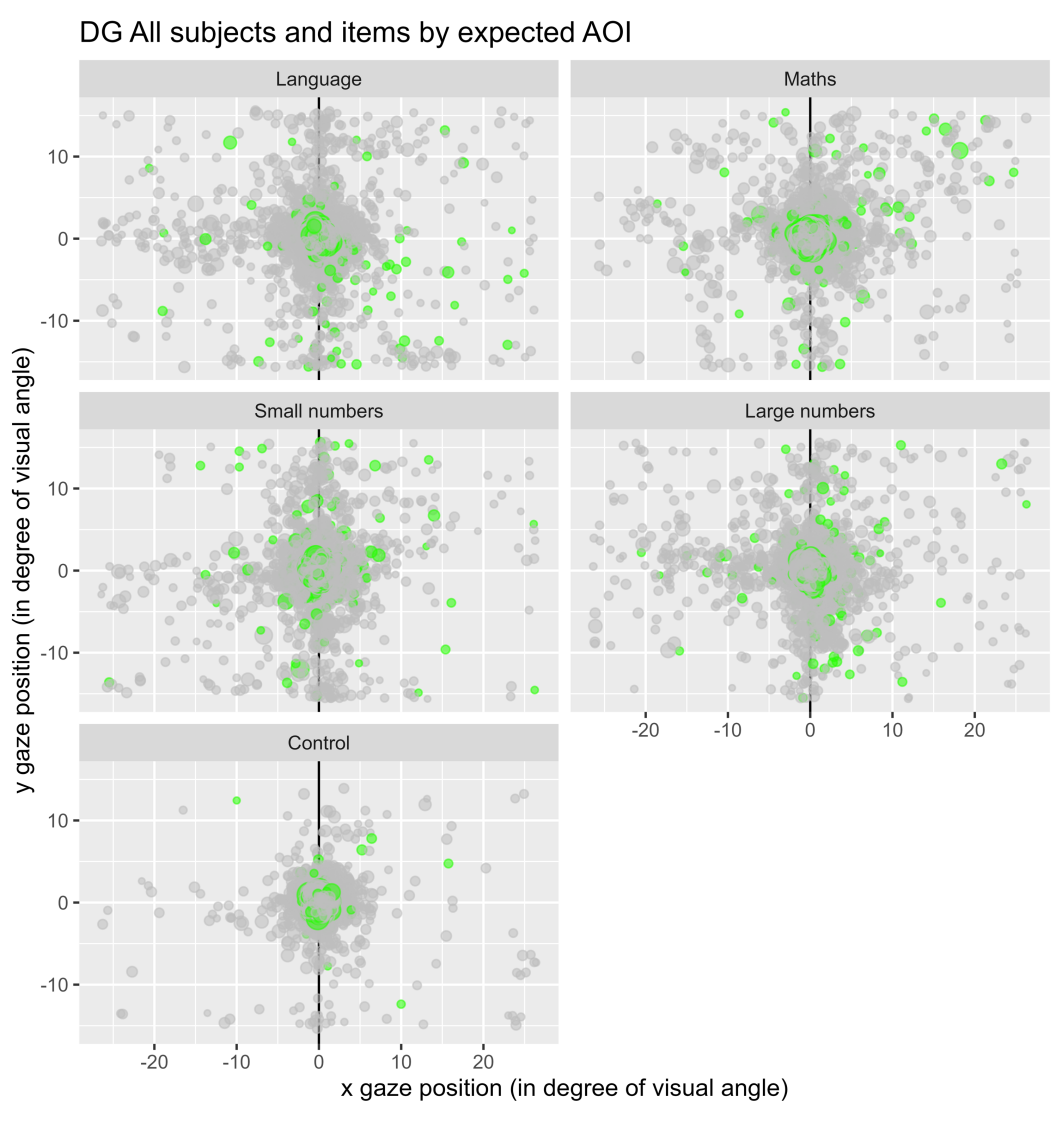
Representation of all the fixations for each condition. Each dot represents a fixation, and the green ones represent the first fixations.

Figure 4 represents the fixations observed for the whole population of participants in the different conditions used. The conditions mathematics (“maths”) and small numbers were expected to induce eye-movements toward the left, while the conditions language and big numbers were expected to induce eye-movements toward the right. Notice that for the control condition, the central zone (area 5) is highly visited compared to the other items.

Table 1 shows the first fixations associated with each zone. The number of fixations in the central zone remains prominent in all conditions. The cells surrounded in red correspond to the expectations as described in the literature.

**Table 1:**
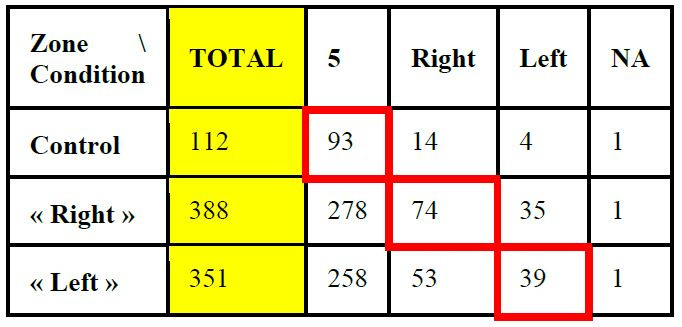
Number of first fixations in each expected AOI.

Notes: Questions inducing language tasks and the representation of big numbers were expected to encourage fixations in the right AOI, while questions inducing space geometry reasoning and the representation of small numbers were expected to encourage fixations in the left AOI. The cells surrounded in red correspond to the literature expectations.

We used a generalized linear mixed-model (GLMM) to determine if the number of fixations in the central (control) area was more important in the control condition than in the other conditions (with a binary discrete variable). We decided to pool all the data obtained from the questions supposed to induce a gaze movement to the right (« imagine a large number » and language tasks) on the one hand, and the questions supposed to induce a gaze movement to the left (« imagine a small number » and spatial geometry tasks), in order to obtain a large number of first fixations. The GLMM results are shown in Table 2.

**Table 2:**
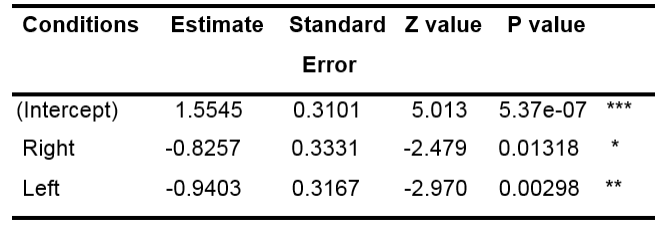
Results of the GLMM model for the first fixations

Note: *p<.05, **p<0.01, ***p<.001

Our test consisted of establishing if the variables “Condition” (type of question asked) and “zone” (visited by the gaze) were dependent or not. The results of the GLMM show that, the estimates of conditions “left” and “right” are lower than the one of the intercepts (i.e. the number of fixations in the center is lower than for the intercept). Moreover, the p-value of the two other conditions is lower than 0.05. Therefore, the number of fixations in the central zone for these two conditions is significantly less important than the number of fixations in the central zone for the control condition.

We also used a GLMM applied to the likelihood of moving the eyes to the right in both right and left conditions. To do so, we chose as discrete variable the probability of right fixation, we removed the control condition and all the fixations in the central area and added a random slope condition by subject. Table 3 illustrates that the probability to fixate the right area in the “left” condition was not significantly different from that of the right condition, contrary to what was expected, suggesting that there was a preference for the right area in the population tested, whatever the condition.

**Table 3:**
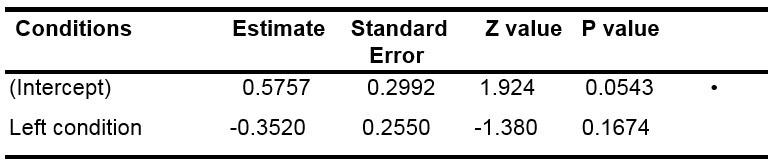
Results of the GLMM model for the first fixations.

Note: • p<.1

**Figure 5:**
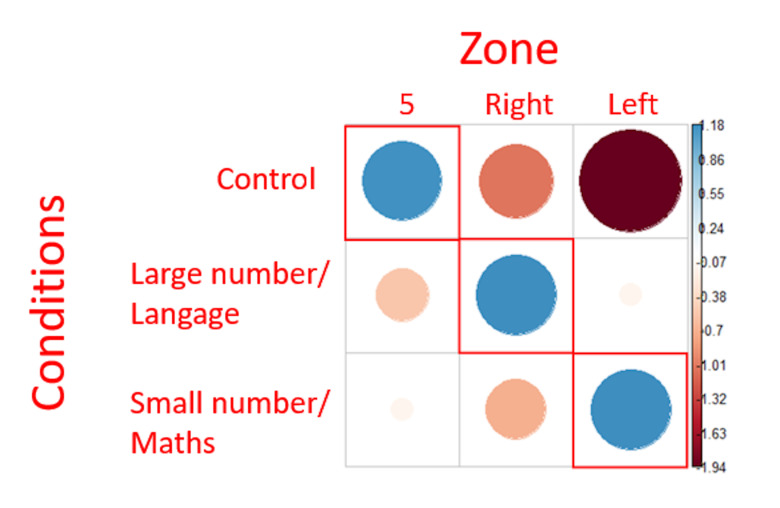
representation of Chi² test residuals for first fixations. Blue circles represent positive relations while red circles represent negative relations. The cells surrounded in red correspond to the theoretical expectations. The diameter of the circles is proportional to the size of the effect.

Nevertheless, the results of the chi² test (Figure 5) show that there is a relationship between the type of question asked and the zone in which the gaze is located as the distribution of all the first fixations is not randomly distributed into the AOIs (X = 9.89, p <.05). This suggests that the zone(s) in which the fixations landed is (at least in part) dependent of the question type.

The positive association observed was only in the expected target zones (Figure 5). This indicates that using our protocol, the preferred gaze orientation revealed by the Chi^2^ analysis corresponded to those described in the literature.

**Figure 6:**
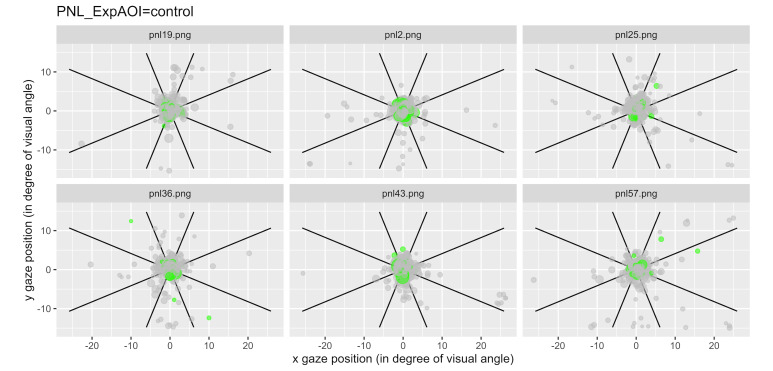
Fixations for each item linked to the control questions for all participants. Each dot represents a fixation, and the green ones represent the first fixations.

**Figure 7:**
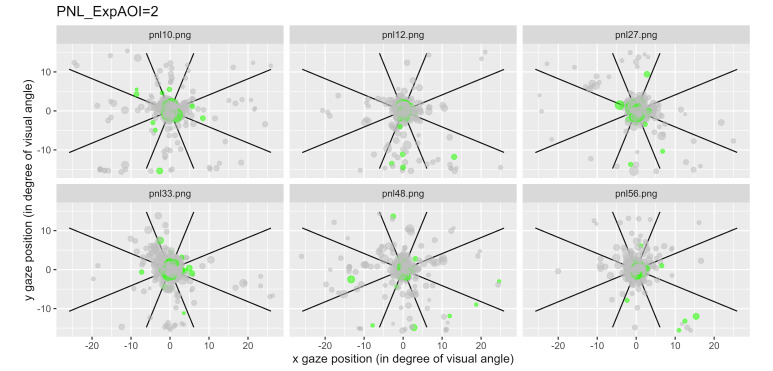
Fixations for each item linked to the expected AOI 2 for all participants. Each dot represents a fixation, and the green ones represent the first fixations.

Results of the NLP experiment:

We have represented the space segmented into nine zones and represented each a gaze fixation by a dot, with the first fixations plotted in green. Figure 6 illustrates examples of typical graphics obtained for the control condition and Figure 7 shows an example of data recorded after questions type 2, for all participant grouped together.

Notice that for the 6 questions of the control condition, the gaze mainly remained in the central area (area 5; Figure 6), while for the 6 questions of type 2, the gaze was more likely to visit areas outside the center area (Figure 7).

In this control condition, the vast majority of fixations remain in the central zone (area 5). If this is true for most of the fixations, and particularly clear for the participants' first fixations which remain very concentrated around the central zone.

Figure 7 illustrates the condition 2 as an example. By comparing Figure 7 to Figure 6, it can be seen that even if the concentration of points remains overwhelmingly in the central zone, there is overall a greater dispersion of the fixations in Figure 7 compared to Figure 6, including first fixations.

Table 4 represents the number of first fixations (black) and the number of total fixations (red) obtained in the different areas, depending on the conditions. The cells surrounded in red correspond to the predictions of the NLP theory.

**Table 4:**
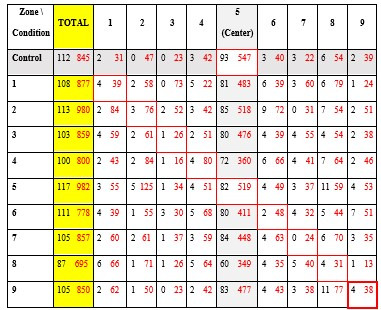
Number of first (black) and total (red) fixations recorded on all participants for each condition and each defined zone.

Note: cells surrounded in red correspond to the predictions of the NLP theory.

Notice that the number of first fixations is very low in order to obtain representative and exploitable results, and a high concentration of fixation in the central zone (minimum of 45% fixation in the central zone for a given condition).

In a first analysis, we used a GLMM with a random effect of condition by subject to determine if there were fewer eye movements in the control condition relative to the other conditions, with the idea that greater mental activity compared to the control condition would induce more gaze movement. We first compared the data obtained for the control questions to those obtained with the other questions to see if there was a difference in gaze movements. We created a binomial variable “is.fix.inside.center” which indicated if each fixation is inside the control area (1) or not (0). Then, we analyzed this variable through a generalized linear regression mixed-model including one fixed effect (i.e., condition) and 2 random effects (i.e., subject and item). The R syntax was the following: glmer(dependent variable ~ condition +(1+condition|Subject) +(1|Item), data=df, family=binomial).

Because the goal of this analysis was to compare the number of fixations inside the control area for the control condition versus each of the other conditions, we defined the control condition as the intercept.

**Table 5:**
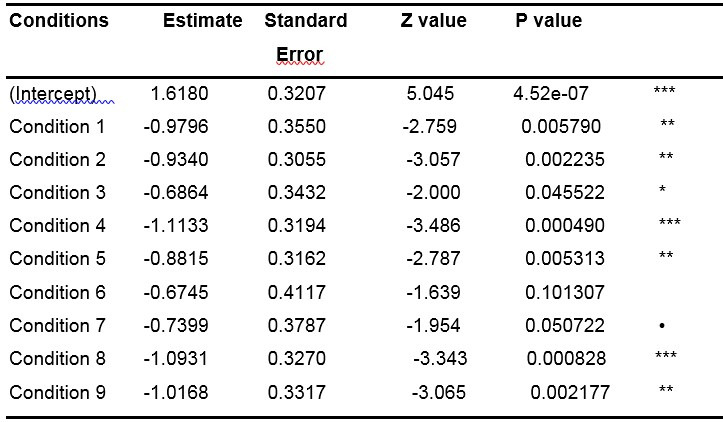
Results of the GLMM (with random slope condition by subject) when analyzing only the first fixations.

Note: ● p<.10, *p<.05, **p<.01, ***p<.001.

The results in Table 5, relative to the "control" condition, (represented by the intercept) suggest that the proportion of the first fixation in the central zone is significantly different from 0 (p-value <.001), which is in line with what we visually observed through the Figures and corroborate the theory. In this table, the Estimates corresponding to all the other conditions are expressed as a function of the intercept (for instance, the Estimate of Condition 1 is equal to 1.6180 - 0.9796 = 0.6384). Z and P values corresponding to each condition specify to what extent the values of the Estimates are significantly different from the Estimate of the intercept.

There are more fixations in the central zone for the control condition than for any other condition (all the estimates of the other conditions are negative, therefore lower than the estimate of the intercept). However, the difference is significant for only a few conditions, namely conditions 1, 2, 3, 4, 5 and 8 (all p-values <.05). This lack of significance for the conditions 6 and 7 (all p-values >.05) could be due to the small size of our data sample regarding first fixations. To verify our idea, we studied the entire range of fixations.

**Table 6:**
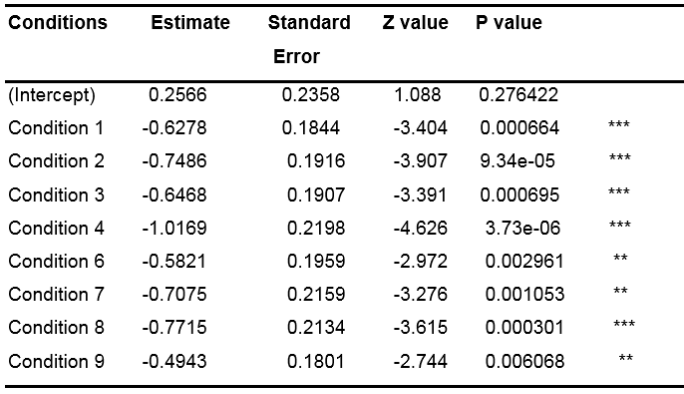
Results of the GLMM (with random slope condition by subject) when analyzing every fixation.

Note: as in Table 5, we can see in Table 6 all conditions are below the value of the Intercept. **p<.01, ***p<.001.

Table 6 shows that every condition contains significantly fewer fixations in the control area than the intercept (i.e., the control condition; all p-values <.01). Therefore, the results of the GLMM analysis show that there are significantly more fixations outside of the center area for each condition compared to the control condition. These results suggest that when some cognitive activity is engaged, people tend to move their eyes more.

The next step was to determine if the type of cognitive activity engaged would determine the gaze direction. Therefore, we computed a second analysis to examine the distribution of the gaze orientation according to the question types.

**Figure 8:**
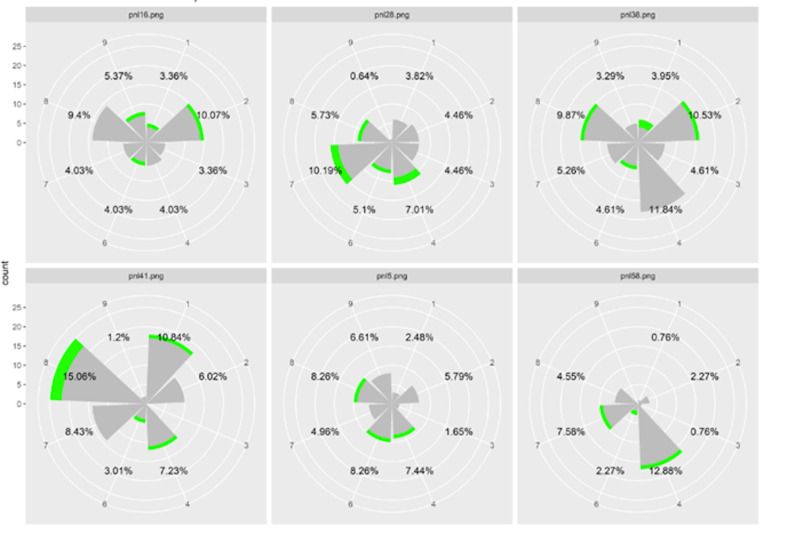
Histograms representing the number of fixations in each zone for each item linked to the expected AOI 1 for all participants. Green represents the number of first fixations in each zone, while grey corresponds to all the subsequent fixations. The difference between 100 and the sum of the percentage corresponds to the number of fixations in zone 5.

In a second analysis, we investigated whether gaze orientations were determined by question types. We observed that within the same question, and within questions of the same condition, there was more than one preferential direction. Figure 8 gives an example of the data obtained with 6 questions of the same type (questions type 1). Each histogram represents the number of fixations in each zone for all participants for each question separately. Percentages are the proportion of fixations in each zone, with zone 5 (center) being excluded. The sum of the percentage in each item is different from 100% because we excluded the zone 5. Notice that the gaze orientation varied among the 6 questions although the questions were designed to be similar (Type 1). Also note that there was more than one preferred direction for most questions.

Figure 8 shows that for each question, the amount of fixation in the center is between 46.04 and 68.93 percent of all fixings. As previously shown in Table 3, it reveals that the number of fixations in the center is preponderant which explains the low percentages for each zone and the heterogeneity of the chosen directions even if some directions stand out very slightly on certain questions (directions 1 and 4).

We, therefore, leaned towards a statistical approach to examine the homogeneity of fixations distribution. Therefore, we proceeded to a Chi² test to determine if the distribution of gaze fixations was purely random or if there were preferential directions. Our test consisted of establishing if the variable “Condition” (type of questions asked) and “zone” (visited by the gaze) were dependent or not.

**Figure 9:**
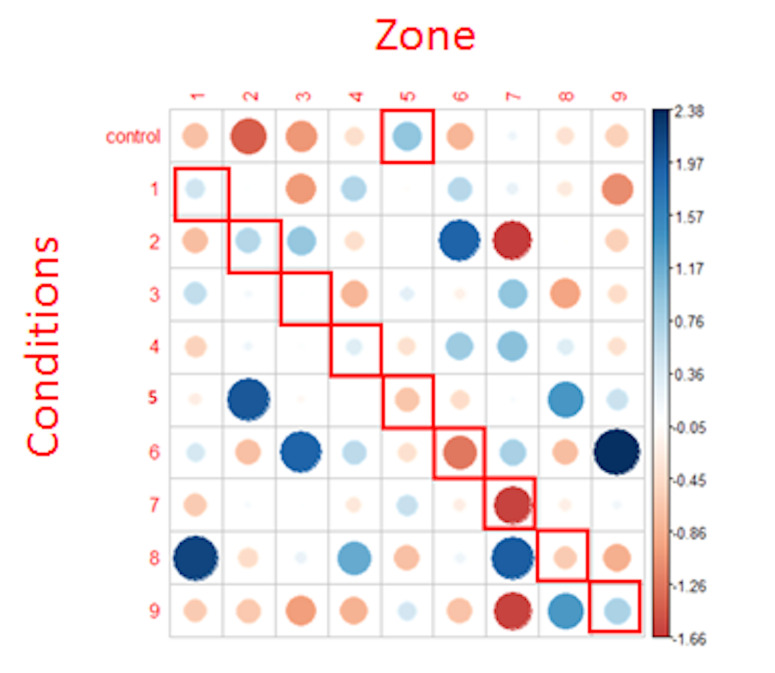
Representation of Chi² test residuals for the first fixations. Blue circles represent positive relations while red circles represent negative relations. The cells surrounded in red correspond to the NLP expectations. The diameter of the circles is proportional to the size of the effect.

The R syntax for this test was as follows: chisq.test(x=condition, y=zone, rescale.p=T, simulate.p.value=T, B=2000)). Residuals of the Chi² test are shown in Figure 9.

Regarding the analysis of the first fixations, the results provided by the Chi² test did not allow us to reject the null hypothesis (X = 67.023, p >.6). In other words, row and column variables were independent, which means that for each condition, fixations were randomly distributed in every AOI. As argued when applying the mixed-models, the number of first fixations in each condition and for each zone is very small, which is likely to have a pernicious impact on the statistical analysis and results.

**Figure 10:**
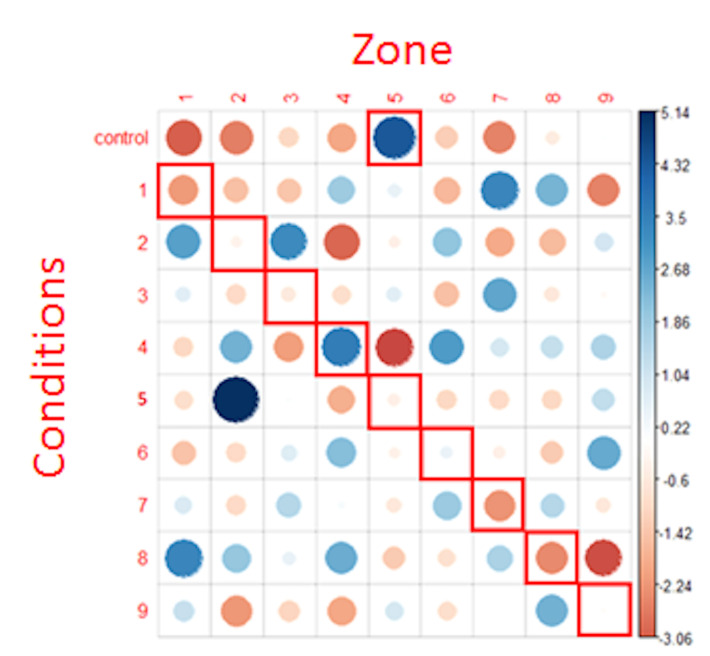
representation of chi² test residuals for all fixations. Blue circles represent positive relations while red circles represent negative relations. The cells surrounded in red correspond to the NLP expectations. The diameter of the circles is proportional to the size of the effect.

We therefore apply a Chi² test after including all the fixations to examine the homogeneity of their distribution. The model applied was the same as the one we used when examining first fixations only. Residuals of this Chi² test are shown in Figure 10.

The results revealed that the distribution of all the fixations was not randomly distributed into the AOIs (X = 318.92, p <.001), thus suggesting that the zone(s) in which the fixations landed (at least in part) depended on the question type.

We can also note some of the associations highlighted by this last graph (for example, control condition and target zone 5, condition 1 and target zone 7, condition 2 and target zones 1 and 3. In particular, the strongest positive association was the anger condition (condition 5) associated with zone 2.

Interestingly, there is no positive association between the conditions and their expected areas according to the NLP hypothesis (cells surrounded in red), except for condition 4, where there is an association with area 4 (but also with areas 2 and 6). In all other conditions, there is rather a neutral or a repulsion effect (red spot) in the predicted area.

## Discussion

In this study, using an original protocol, we showed that simple questions eliciting little cognitive activity (“control” questions) induced significantly fewer eye movements compared to questions that were more complex. We found that the eye movements were not random according to the different question types eliciting different thought processing but oriented in selected directions. Most of the time, the orientations we observed were not those predicted by the NLP theory but were subject-specific, as described in Burke et al. (5).

Using analysis on videotapes, Farmer et al. (11) had already shown that studies supporting the NLP hypothesis on eye movement related to different sensory modalities were likely biased because of the choice of statistical analysis. Our study using eye tracking removes the bias of the observer as a machine made the measurements. Data driven approaches with a larger set of data could bring new insights in the future.

### Limitations

The choice of the number of participants was based on studies that used eye trackers in other contexts than NLP theory (4, 10, 12, 14) because this particular hypothesis of NLP had never been tested with an eye tracking system. We did the experience with 31 subjects but had to remove data from some of them. The results of the present study have therefore to be considered with caution, as a pilot study. In addition, the participants were not fully representative of the general population and not diverse enough: our sample was mainly composed of women (thus there may be a gender bias), and of academic people and we only tested right handed participants.

Another problem concerns the participant's freedom of movement. At the beginning of the tests we did two calibrations: a general one with the Tobii Manager software and a second one just before recording with Tobii Pro. However, the calibration takes into account not only the distance of the subject from the screen but also the position of the subject's head. For the data to be correct, the subject should remain motionless throughout the experiment. In our experiment, to remain in an ecological situation, participants were free to move (no chinstrap or landmarks where to place their heads, etc). Over a cumulative 30 min of testing, we can easily assume that people did not remain static throughout the experiment. This could have affected the accuracy of the measures, linked to the calibration, as the subjects could have slightly modified their position (distance to the screen or slight shift of the head) during the experiment. While the choice not to hinder the participants’ movements was very important to us in this experiment (we wanted the subject to feel as little as possible in an experiment and therefore to react as "naturally" as possible), it cannot be denied that the consequences of this choice were far from negligible.

We also observed different behaviors of the participants that could be problematic for our data: first, the preponderance of fixations at the center can be explained by the fact that subjects thought they had to return to the center systematically at the end of the question. If we add this to the phenomenon of retinal persistence due to the fixation cross during the question (that we tried to lower as much as possible using a low luminance of the cross), we can assume that the subjects were perhaps conditioned to remain in the center (consciously or not). Using small spots moving circularly in the center of the screen might improve the acquisition.

Trying to compensate for the subject's movements while continuing to observe the eye movements, it would potentially be possible to conduct a similar experiment with a virtual reality helmet equipped with an eye tracker. The advantage would be double since we would always have the position of the gaze while allowing the subject to remain free of his movements without affecting the results (the gyroscopic system of a VR helmet will take into account the head movements made by the subject). In addition, the gaze of the participants often went out of the screen and Tobii was unable to cope with the gaze outside the screen area, thus we got a lot of missing data and had to use not only the first fixation, but all the fixations in the 5 sec duration after the cross disappeared from the screen. Using a VR helmet could solve this problem.

In itself, the participants’ task was simple as they were “just” answering a question. It might be interesting to adapt our protocol to see what their eye behavior would be in another setting (full interaction with another participant for example) to try to tend as much as possible towards a more natural behavior and experiment. The participant would feel less in an experiment and would operate as naturally as possible, without feeling like being in an experiment.

We also observed that some participants always had the same preferential direction regardless of the type of question asked. We considered this bias by using a GLMM using random effects of condition by subjects.

Another limitation/question is the choice of the AOIs used. We have defined these as arbitrary strips and it may be that this dimension does not represent reality. Should we keep the equitable aspect of each zone (same angle spacing for each zone) or should we modify these different parts? Should we pool the results obtained in two closest AOIs? A data driven approach could bring new insights in the future.

Finally, one might wonder about the very essence of the tests: the questions. Indeed, we started from the hypothesis that our questions were supposed to correspond to one and only one zone. However, in the thinking process, it is quite likely that a question triggered different cognitive processes depending on the participant (for instance, if we asked a question about anger, the participant would possibly visually remember a scene, which implies a cognitive process that is quite different). We also asked ourselves whether the ambiguity of certain questions could possibly be considered (to estimate which cognitive process would be elicited by the various questions and therefore which potential target area should be expected). A phenomenological study could help us trace the thought process used by the participants to try to answer the question asked. The level of difficulty of the questions is also a point to keep in mind: during the tests for instance, we asked the participants to imagine on some questions extremely small numbers or to give the distance between earth and moon. The tasks seemed difficult to some of them. Do the participants try to imagine or find the value, or do they disengage the cognitive process, as the task seems too difficult for them? The cognitive process involved is probably different (a similar problem to the one regarding the ambiguity of the questions). How could we take into account the difficulty of a task?

We also encountered technical problems during the experiment and thus got some missing data because some subjects either closed their eyes or because their gaze came out of the screen at certain times, which was not properly treated by the Tobii system.

The results we have presented here are based on all participants, but perhaps the gender of the participant had an impact on the results. It will be interesting to compare the results to those of left-handed participants. We also did not take into account the common specificities of the participants and data mining could provide us with new results or avenues for reflection.

Finally, our experimental protocol did not fully replicate the right/left experiment described in the literature (8; 14) as there was a preference for the right area in the population tested. Non-confirmation of NLP expectations should therefore be taken with caution.

### Conclusions

The results of this study show that there are more eye movements in response to questions that require more mental activity than in response to control questions requiring less mental activity. The study goes even further, by showing that for each type of question, there are one or more preferential directions correlated with the type of question. However, these directions are different from those claimed by the NLP theory. We evaluated our protocol by a control experiment but we did not completely replicate the data from the literature. Therefore, the present results should be taken with utmost caution. This is likely due to the low number of participants and the small number of reliable first fixations recorded. To get around this obstacle, we studied the results obtained in all the fixations recorded following the end of the question during 5 seconds, which is not exactly what we planned to do at the beginning. Overall, this pilot study describes an original methodology that could be useful for further research in this field. It paves the way for other experiments that could associate the eye tracker in a virtual reality helmet with micro-phenomenology in order to determine more precisely the intellectual path that the individual took to answer the question and thus compare more precisely its performances to the presuppositions of NLP on gaze, or even redefine a new theory.

### Ethics and Conflict of Interest

The author(s) declare(s) that the contents of the article are in agreement with the ethics described in http://biblio.unibe.ch/portale/elibrary/BOP/jemr/ethics.html and that there is no conflict of interest regarding the publication of this paper.

The protocol was approved by the Ethic Committee of University Côte d’Azur (CERNI), N° 2020-4-003 (Supplementary data 4).

Scripts are available on demand.

### Acknowledgements

The authors would like to thank all the volunteers that participated this study. The authors greatly acknowledge the CocoLab (Complexity and Cognition Lab, MSHS Sud- Est (USR3566), Université Côte d’Azur - CNRS) and its staff, particularly Ambre Denis Noël for her advices and help in the statistics and Jean Charles Briquet Laugier for logistic. We thank Laurent Michau (Psycho-practitioner therapist in brief therapies: hypnosis and NLP) for his advices on the protocol. We thank Olivia Vidal and Ilan Sansoni for their help during the experiments and Patricia Bouret for her statistical advice.

This work was supported by the French government through the UCA-Jedi project managed by the National Research Agency (ANR-15- IDEX-01) and, in particular, by the interdisciplinary Institute for Modeling in Neuroscience and Cognition (NeuroMod) of the University Côte d’Azur. Mathieu Marconi was funded by Neuromod Institute of University Cote d’Azur.

## supplementary material



## References

[b1] Bandler, R., & Grinder, J. (1975a). The Structure of Magic I : A Book About Language and Therapy. Science & Behavior Books.

[b2] Bandler, R., & Grinder, J. (1975b). The Structure of Magic II : A Book About Communication and Change. Science & Behavior Books.

[b3] Bandler, R., & Grinder, J. (1976). Patterns of the Hypnotic Techniques of Milton H. Erickson, M.D (Vol. I). Meta Publications.

[b4] Buckner, M., & Meara, N. M. (1987). Eye Movement as an Indicator of Sensory Components in Thought. Journal of Counseling Psychology, 34(3), 283–287. 10.1037/0022-0167.34.3.283 1939-2168

[b5] Burke, D. T., Meleger A., & Schneider J. C. (2003). Eye-movements and ongoing task processing. Perceptual and Motor Skills, 96(3), 1330-1338. 10.2466/pms.96.3.1330-1338 10.2466/pms.96.3.1330-1338 12929791

[b6] Canadian Agency for Drugs and Technologies in Health. (2014). Neuro-Linguistic Programming for the Treatment of Adults with Post-Traumatic Stress Disorder, General Anxiety Disorder, or Depression: A Review of Clinical Effectiveness and Guidelines. https://pubmed.ncbi.nlm.nih.gov/25473689/25473689

[b7] Davidson, P. R., & Parker, K. C. (2001). Eye movement desensitization and reprocessing (EMDR): A meta-analysis. Journal of Consulting and Clinical Psychology, 69, 305–316. 10.1037/0022-006X.69.2.305 0022-006X 11393607

[b8] Dehaene, S. (2010). La bosse des maths. Odile Jacob.

[b9] Dooley, K. O. D., & Farmer, A. (1988). Comparison for Aphasic and Control Subjects of Eye Movements Hypothesized in Neurolinguistic Programming. Perceptual and Motor Skills, 67(1), 233-234. 10.2466/pms.1988.67.1.233 3211676

[b10] Elich, M., Thompson, R. W., & Miller, L. (1985). Mental Imagery as Revealed by Eye Movements and Spoken Predicates: A Test of Neurolinguistic Programming. Journal of Counseling Psychology, 32(4), 622–625. 10.1037/0022-0167.32.4.622 1939-2168

[b11] Farmer, A., Rooney, R., & Cunningham, J. R. (1985). Hypothesized Eye Movements of Neurolinguistic Programming : A Statistical Artifact. Perceptual and Motor Skills, 61(3), 717-718. 10.2466/pms.1985.61.3.717 4088761

[b12] Galin D., & Ornstein R. (1974). Individual differences in cognitive style –I. Reflective eye movements, Neuropsychologla, 12, 367-376. 10.1016/0028-3932(74)90052-9 4425336

[b13] Karunaratne, M. (2010). Neuro-linguistic programming and application in treatment of phobias. Complementary Therapies in Clinical Practice, 16, 203–207. 10.1016/j.ctcp.2010.02.003 1873-6947 20920803

[b14] Kocel, K., Galin, D., Orntein, R., & Merrin, E. L. (1972). Lateral eye movement and cognitive mode. Psychonomic Science, 27(4), 223–224. 10.3758/BF03328944 0033-3131

[b15] Mehrabian, A., & Ferris, S. R. (1967). Inference of attitudes from nonverbal communication in two channels. Journal of Consulting Psychology, 31(3), 248–252. 10.1037/h0024648 0095-8891 6046577

[b16] Mehrabian, A., & Wiener, M. (1967). Decoding of inconsistent communications. Journal of Personality and Social Psychology, 6(1), 109–114. 10.1037/h0024532 0022-3514 6032751

[b17] Novo Navarro, P., Landin-Romero, R., Guardiola-Wanden-Berghe, R., Moreno-Alcázar, A., Valiente-Gómez, A., Lupo, W., García, F., Fernández, I., Pérez, V., & Amann, B. L. (2018). 25 años de Eye Movement Desensitization and Reprocessing: Protocolo de aplicación, hipótesis de funcionamiento y revisión sistemática de su eficacia en el trastorno por estrés postraumático. Revista de Psiquiatría y Salud Mental, 11(2), 101–114. 10.1016/j.rpsm.2015.12.002 10.1016/j.rpsm.2015.12.002 2173-5050 26877093

[b18] Pluzyczka, M. (2018). The First Hundred Years: A History of Eye Tracking as a Research Method. Applied Linguistics Papers, 25(4), 101–116. 10.32612/uw.25449354.2018.4.pp.101-116 2544-9354

[b19] Poffel, S. A., & Cross, H. J. (1985). Neurolinguistic programming: A test of the eye-movement hypothesis. Perceptual and Motor Skills, 61(3 Pt 2, suppl), 1262. 10.2466/pms.1985.61.3f.1262 0031-5125 4094868

[b20] Rajeswari Sreelekha, H. B. (2017). Effectiveness of Neurolingiuistic Programming (nlp) On Secondary Traumatic Stress (sts) Among Nurses, GJRA - Global Journal For Research Analysis(GJRA) | World Wide Journals. https://www.worldwidejournals.com/global-journal-for-research-analysis-GJRA/.https://www.worldwidejournals.com/global-journal-for-research-analysis-GJRA/article/effectiveness-of-neurolingiuistic-programming-nlp-on-secondary-traumatic-stress-sts-among-nurses/NzA5Mg==/?is=1

[b21] R Core Team R. (2020). A language and environment for statistical computing. R Foundation for Statistical Computing, Vienna, Austria. URL https://www.R-project.org/

[b22] Sharpley, C. F. (1984). Predicate matching in NLP: A review of research on the preferred representational system. Journal of Counseling Psychology, 31(2), 238–248. 10.1037/0022-0167.31.2.238 1939-2168

[b23] Sturt, J., Ali, S., Robertson, W., Metcalfe, D., Grove, A., Bourne, C., & Bridle, C. (2012). Neurolinguistic programming: A systematic review of the effects on health outcomes. The British Journal of General Practice, 62, e757–e764. 10.3399/bjgp12x658287 10.3399/bjgp12x658287 1478-5242 23211179 PMC3481516

[b24] Thomason, T. C., Arbuckle, T., & Cady, D. (1980). Test of the eye-movement hypothesis of neurolinguistic programming. Perceptual and Motor Skills, 51(1), 230. 10.2466/pms.1980.51.1.230 0031-5125 7432961

[b25] Tosey, P., & Mathison, J. (2007). Fabulous creatures of HRD: a critical natural history of neuro-linguistic pro-gramming In: Paper presented at the 8th international conference on human resource development research and practice across Europe, Oxford Brookes Business School.

[b26] Vranceanu, R., Vertan, C., Condorovici, R., Florea, L., & Florea, C. (2011). A fast method for detecting eye accessing cues used in Neuro-Linguistic Programming. 2011 IEEE 7th International Conference on Intelligent Computer Communication and Processing.

[b27] Wertheim, E. H., Habib, C., & Cumming, G. (1986). Test of the Neurolinguistic Programming Hypothesis That Eye-Movements Relate to Processing Imagery. Perceptual and Motor Skills, 62(2), 523-529. 10.2466/pms.1986.62.2.523 3503261

[b28] Wiseman, R., Watt, C., ten Brinke, L., Porter, S., Couper, S.-L., & Rankin, C. (2012). The eyes don’t have it: Lie detection and Neuro-Linguistic Programming. PLoS One, 7(7), e40259. 10.1371/journal.pone.0040259 1932-6203 22808128 PMC3394779

[b29] Yarbus, A. L. (1967). Eye Movements and Vision. Plenum Press. 10.1007/978-1-4899-5379-7

[b30] Zaharia, C., Reiner, M., & Schütz, P. (2015). Evidence-based neuro linguistic psychotherapy: A meta-analysis. Psychiatria Danubina, 27(4), 355–363.0353-5053 26609647

