# Supplementary material for: Eye movements in response to different cognitive activities measured by eyetracking: a prospective study on some of the neurolinguistics programming theories
Source: J Eye Mov Res. 2023 May 16;16(2):10.16910/jemr.16.2.2. doi: 10.16910/jemr.16.2.2 (PMC10676768; doi:10.16910/jemr.16.2.2)
Supplement: Supplementary file 1 [file jemr-16-02-b-SD1-01.pdf]

Supplementary data

1- Consent for participation

CONSENTEMENT DE PARTICIPATION

TITRE de L'ETUDE : Etude des expressions faciales par une méthode non invasive en réponse à diverses questions.
Promoteur : CNRS, IPMC
Investigateur Principal : Alice Guyon alice.guyon@ipmc.cnrs.fr, 0618721100

PERSONNE(S) QUALIFIEE(S) :
Nom de l'Investigateur : Mathieu Marconi
Nom de l'Investigateur Principal : Alice Guyon
....
Je confirme avoir expliqué en détail l'étude référencée ci-dessus. J'ai répondu à toutes les questions qui m'ont été posées concernant l'étude.

Date : ....

Signature de la personne recueillant le consentement :

SUJET DE L'ETUDE :
Nom du sujet : .... Prénom : ....
Adresse :
....
....
....
Tél. Domicile : .... Tél. Portable :
....
Email :
....
Je reconnais avoir reçu toutes les explications concernant l'étude : son but, son déroulement, ses bénéfices/risques potentiels ainsi que mes obligations (déclaration des données et respect du protocole), mes conditions de participation et mes droits.
J'ai pu poser toutes mes questions avant de prendre ma décision et j'ai bénéficié d'un délai de réflexion suffisant avant de signer ce consentement de participation.
Je sais que j'ai la possibilité de demander des informations complémentaires à l'investigateur ou à ses représentants.
J'accepte de participer à ce test librement, sans aucune sorte de pression. Je sais que je pourrai décider à tout moment d'interrompre le test sans aucun préjudice pour moi. J'en informerai alors l'investigateur ou ses représentants.
J'ai également été prévenu(e) que l'investigateur et/ou le promoteur peuvent interrompre l'étude pour certaines raisons (événement indésirable jugé sévère, violations majeures au protocole, etc.).
J'accepte que les données personnelles enregistrées à l'occasion de cette étude puissent faire l'objet d'un traitement informatisé, pour le compte du promoteur. J'ai compris que selon les dispositions de la loi relative à l'informatique, aux fichiers et aux libertés, j'ai un droit d'accès et de rectification des données (s'adresser à Alice Guyon). Je pourrai également exercer mon droit d'opposition à la transmission des données couvertes par le secret professionnel susceptibles d'être utilisées et d'être traitées dans le cadre de cette recherche. Si je fais usage de ce droit d'opposition à la transmission, cela aura pour conséquence une impossibilité de participer à l'étude.
Je sais que toutes mes données seront traitées avec la plus grande confidentialité.
Je consens également à un archivage de mes données (vidéos, réponses aux questions) de manière confidentielle (sans aucune indication de mon nom). Le respect de la confidentialité sera basé sur la pseudonymisation et une table de correspondance non accessible en dehors des personnes identifées.

Mon consentement ne décharge pas le promoteur et l'investigateur de leurs obligations légales.
Je conserve tous mes droits garantis par la loi. J'ai reçu, ainsi que l'investigateur, un original de ce consentement. J'ai également reçu un original de la fiche d'informations et signé une feuille d'émargement le stipulant.
Dans ces conditions, j'accepte de participer à cette étude et je confirme répondre aux critères standards d'inclusion et de non-inclusion, listés ci-dessous :

- Disponible pour la totalité de la durée de l'étude
- Etant motivé(e) et libre de participer à l'étude
- S'engageant à suivre la procédure
- Comprenant la langue française: sujet francophone capable de lire les documents qui lui sont présentés, d'adhérer à ce qui lui est expliqué et de compléter les formulaires d'étude en français.
- Ne pas être mineur(e) (article L1121-7) ou être un individu majeur bénéficiant d'une mesure de protection légale ou étant incapable d'exprimer son consentement (article L1121-8) dès lors que la recherche peut être réalisée autrement

Date : ....

Signature du sujet, précédée de la mention « Lu et approuvé » :
....

2- Information form

FICHE D'INFORMATION

TITRE de L'ETUDE :
**Etude des expressions faciales par une méthode non invasive en réponse à diverses questions.**
**Promoteur : CNRS, IPMC**
**Investigateur Principal : Alice Guyon alice.guyon@ipmc.cnrs.fr, 0618721100**

Promoteur : CNRS, IPMC

Investigateur Principal : Alice Guyon alice.guyon@ipmc.cnrs.fr, 0618721100

PERSONNE(S) QUALIFIEE(S) :

Nom de l'Investigateur : Mathieu Marconi Nom de

l'Investigateur Principal : Alice Guyon Madame,

Monsieur,

Nous réalisons une étude scientifique afin d'évaluer les effets de l'activité cérébrale sur les expressions faciales. Pour cette étude, nous allons recruter une quarantaine de participants volontaires, hommes, femmes adultes entre 18 et 60 ans.

Les sujets volontaires seront assis devant un écran et devront répondre à des questions délivrées par un casque audio. Ils seront filmés durant toute la séance de façon à évaluer les effets de questions sur leurs expressions faciales.

L'ensemble de l'étude prendra environ 40 minutes pour chaque sujet. Les données resteront strictement confidentielles et ne serviront qu'à l'étude. Les données seront analysées automatiquement par un logiciel. L'objectif principal de l'étude vise à déterminer les liens possibles entre activité cérébrale et expressions faciales selon les observations empiriques réalisées par les praticiens de la PNL (programmation Neuro-linguistique).

Les expériences auront lieu sur la plateforme CoCoLab, Maison des Sciences de l'Homme et de la Société Sud-Est, (24, avenue des Diables Bleus, Campus Saint Jean d'Angély, 06100 Nice).

L'étude sera menée par Mathieu Marconi, étudiant en Master of Sciences « Modelisation for neuroscience and cognition », MOD4NEUCOG, supervisé par Alice Guyon, Directrice de Recherches au CNRS (IPMC).

Les participants prendront connaissance de l'étude avant l'expérience et signeront un formulaire de consentement éclairé en deux exemplaires avant l'expérience (l'un remis à l'expérimentateur, l'autre conservé par le volontaire).

3- Question list (French/English)

The list of questions used in the present study is available at (French questions and English translation):

https://nuage.ipmc.cnrs.fr/s/QAM7NmWMN9cqomj

4- Ethic committee

UNIVERSITÉ CÔTE D'AZUR

Comité d'Éthique pour les Recherches Non Interventionnelles

(CERNI)

AVIS n° 2020-4-003

Le CERNI, après en avoir délibéré, émet un avis favorable au Projet intitulé « Evaluation scientifique des liens potentiels entre activité cérébrale et mouvements des yeux »

Responsable scientifique : GUYON Alice, Directeur de recherche (DR2) au CNRS, IPMC (Institut de Pharmacologie moléculaire et Cellulaire).

Il est rappelé que le présent avis est délivré sans être exclusif des éventuelles démarches visant à la protection des données personnelles à réaliser en interne (délégué à la protection des données (DPO) de l'établissement : M. Didier Martin, Didier.MARTIN@univ-cotedazur.fr).

Fait pour valoir ce que de droit à Nice, le 04 mars 2020.

Prof. Doct. Yves STRICKLER
Référent éthique et intégrité scientifique
*Président du CERNI (*Comité éthique pour les recherches non interventionnelles*)*

Université Côte d'Azur - Grand Château - 28 avenue de Valrose - 06103 Nice Cedex 2

5-
